# Advances in the Rehabilitation of Hemispatial Inattention

**DOI:** 10.1007/s11910-023-01252-8

**Published:** 2023-03-04

**Authors:** Neena R. Singh, Alexander P. Leff

**Affiliations:** 1 UCL Queen Square institute of Neurology, University College London, London, UK; 2 Department of Neurology, Neurosciences Institute, Cleveland Clinic London, London, UK

**Keywords:** Hemispatial inattention, Neglect, Sensory stimulation, Brain stimulation, Dopaminergic therapy, Rehabilitation

## Abstract

**Purpose of Review:**

There continue to be a plethora of approaches to the rehabilitation of hemispatial inattention, from different forms of sensory stimulation (visual, auditory and somatosensory feedback), through all major modes of non-invasive brain stimulation to drug therapies. Here we summarise trials published in the years 2017–2022 and tabulate their effect sizes, with the aim of drawing on common themes that may serve to inform future rehabilitative studies.

**Recent Findings:**

Immersive virtual reality approaches to visual stimulation seem well tolerated, although they have yet to yield any clinically relevant improvements. Dynamic auditory stimulation looks very promising and has high potential for implementation. Robotic interventions are limited by their cost and are perhaps best suited to patients with a co-occurring hemiparesis. Regarding brain stimulation, rTMS continues to demonstrate moderate effects but tDCS studies have yielded disappointing results so far. Drugs, primarily aimed at the dopaminergic system, often demonstrate beneficial effects of a medium size, but as with many of the approaches, it seems difficult to predict responders and non-responders.

**Summary:**

Our main recommendation is that researchers consider incorporating single-case experimental designs into their studies as rehabilitation trials are likely to remain small in terms of patient numbers, and this is the best way to deal with all the factors that cause large between-subject heterogeneity.

## Introduction

Hemispatial inattention, also known as ‘neglect’, is an acquired neurological deficit affecting attention with a bias towards one side of space. This is usually manifested in extra-personal space but can also affect the patient’s own body parts or intra-personal space. This causes significant disability and, when stroke is the cause, often reduces the effectiveness of attempts to rehabilitate co-occurring deficits such as hemiparesis, causing increased in-patient stays and reduced functional independence [1]. It occurs in 25–30% of people hospitalised by a stroke and despite a degree of spontaneous recovery it can persist into the chronic phase (>3 months) in a third of cases [2].

Given its significant impact on stroke recovery, there has been a considerable focus on the rehabilitation of hemispatial inattention, with a variety of approaches trialled over the past 60 years employing either sensory stimulation, indirect brain stimulation, or drugs. Here we review the most recent studies in the field. Given the diversity of approaches, it can be challenging to compare results across studies. This is compounded by the lack of standardisation of outcome measures (summarised in Table 1). We have addressed this by tabulating unstandardised (raw) and standardised (usually Cohen’s *d*) effects for all the reviewed studies, where these have been reported or can be calculated from the data or figures provided (Table 2). We have limited ourselves to primary outcome measures in the main text and split these into measures of impairment (e.g. cancellation tasks) or function (e.g. the Catherine Bergego Scale (CBS)). There is no accepted Minimally Important Clinical Difference (MICD) for cancellation tasks. For the CBS, a reduction of four points is considered meaningful [3].

**Table 1.**
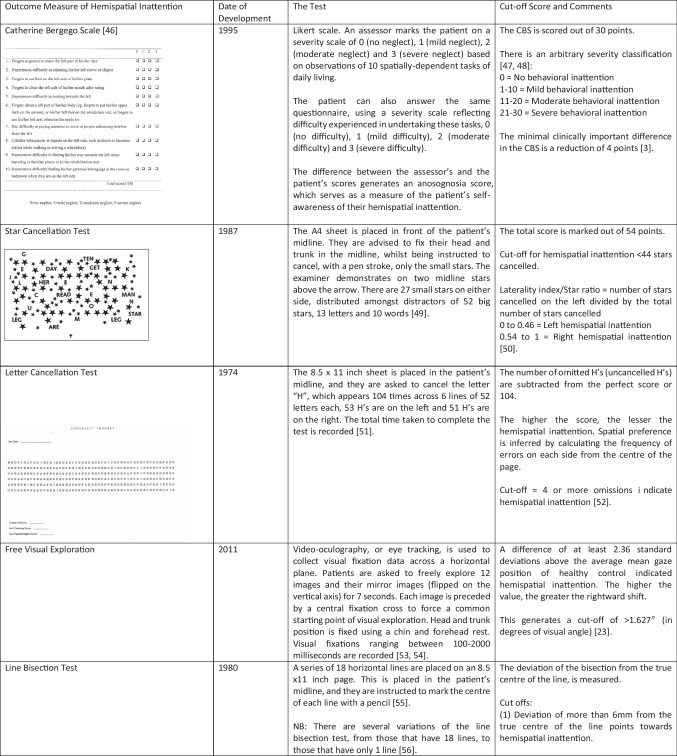

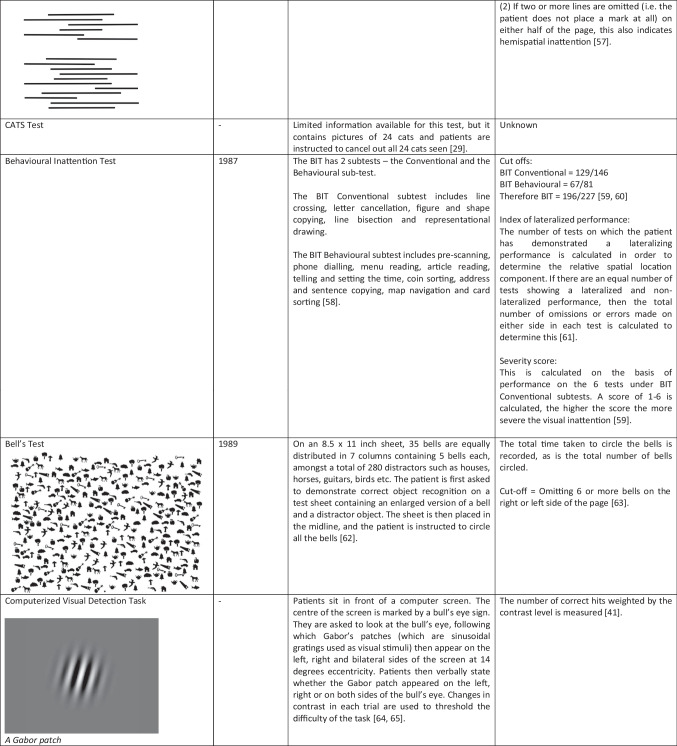
Summary of the key outcome measures employed by the studies discussed in the main text. Where available, clinical cut-offs and minimally important clinical difference scores are provided

.

**Table 2:** Summary of the participants, study design, intervention, outcome measures and effect sizes of the papers discussed in the main text.

	Author, year	Subjects in therapy group	Drug and training interventions	Key outcome measures	Significant differences; effect sizes, raw and standardised (Cohen’s *d*)
*Visual scanning and congruent movement training*	Elshout et al. 2019	**Single-blind, group randomised CMT group** *N* = 15, age = 59.2, TSS = 102.6 days **Visual scanning training** *N* = 15, age = 58.7, TSS = 76.8 days	Congruent movement training (simultaneous eye and hand movements to same location in the affected hemifield) 10 sessions of training, 30min per session, parallel to standard rehabilitation programme. VST control group. Patients instructed to make eye movements to the affected hemifield to detect a specific stimulus	Shape cancellation Line bisection task Catherine Bergego Scale	**Composite score across two cancellation tasks and CBS** *CMT group* Mean difference = −5.8 points *VST group* Mean difference = +2.5 points Cohen’s *d* = 0.53 (medium)
*Immersive HMD VR*	Yasuda et al. 2017	**Within-subject, order randomised, pre- and post-design with no control or blinding** *N*=10, age=70.6, TSS= 149.3 days All patients had both “near” and “far” neglect	Oculus Rift DK2 with Leap Motion, Unity 5, far and near space training 30-min session with each tasking running for 4 min at a time with an interval of 30-s rest between them	Line cancellation task Star cancellation task Line bisection task Letter cancellation task Timepoints: Pre and immediately post test	**Star cancellation (median)** Near group difference = −3 stars Far group difference = +2 stars
*Immersive HMD VR*	Choi et al. 2021	**Single-blind, randomised controlled Digital practice group** *N*=12, age = 63, TSS= 4.33 months **Control group** *N*=12, age = 61.58, TSS= 4.58 months	Oculus Rift with Leap Motion, 10 different tasks (blocks, element L, warlock, laser, pinch draw, RPS island, VT table tennis). 4-week practice programme, 3 sessions/week, a half-hour/session Control group underwent conventional USN specific training for 30 min, 3 times a week for 4 weeks, for total of 12 sessions	Line bisection test Catherine Bergego Scale Modified Barthel Index Motor free visual perception test vertical version Head rotation Timepoints: Pre and post test	**Catherine Bergego Scale:** No significant between group effect.
*Audio*	Kaufmann et al. 2022	Experiment 1 (looking at short term effects of auditory stimulation with and without auditory spatial cueing) **Cross over, repeated measures, randomised and single-blinded** *N*=9, age=64.78, TSS=22.67 days Experiment 2 (looking at 1 and 3 h aftereffects of auditory stimulation with and without auditory spatial cueing) **Single-blinded, repeated-measures, cross-over design** *N*=12, age=73.17, TSS=19.08 days	Experiment 1: listening to preferred music with or without auditory spatial cuing for 10 min, once a day, 2 days. Experiment 2: listening to preferred music with or without auditory spatial cuing for 15 min, once a day, 2 days.	Experiment 1: Letter cancellation test Experiment 2: Free view exploration All the patients in 2 experiments:Voxel based lesion symptom analysis	**Experiment 1** *With auditory spatial cueing:* Mean change = −0.264, SD = 0.237; *Without auditory spatial cueing:* Mean change = −0.106, SD = 0.107; Cohen’s *d* = 0.85 (large) **Experiment 2** Mean gaze position between both groups at the 1-h time point *p*=0.500 Mean gaze position with spatial cueing group at 3-h time point *p*=0.045 eta square = 0.039
*Audio and multisensory (audio+OKS)*	Schenke et al. 2021	Experiment 1 (dynamic auditory cueing) **Double-blinded, historical control group** *N*=11, age = 69.2, TSS= 35.4 days Control group *N*=14, age= 67, TSS=42.1 days Experiment 2 (multisensory) **Double-blinded, no control** *N*=8, age = 59.8, TSS= 36.5 days	15 sessions of 30 min over 3 weeks Experiment 1: Dynamic auditory cuing Experiment 2: Combined therapy dynamic auditory cues and optokinetic stimulation (patients observe contralateral motion of visual patterns or targets on a screen and execute active and smooth pursuit eye movement towards the direction of the motion) Control group had general neuropsychological therapy	Experiment 1: Line bisection test Apple test Timepoints experiment 1: Baseline T1 (day 1), T2 (day 4—just before intervention), T3 (day 25—post 3 weeks of therapy), T4 (day 28—intervention group only) Experiment 2: Visual scanning of the test battery for attentional performance Timepoints experiment 2: Baseline T1 and T2, then T3 post intervention	Experiment 1: **Line bisection test** Between group effect size at T1–T3 Cohen’s *d* = 0.38 (small) Experiment 2: **Mean number of omissions on the left** T1 to T2 Cohen’s *d* = 0.39 (small) T1 to T3 Cohen’s *d* = 2.25 (huge)
*Multisensory—audio visual versus PA*	Zigiotto et al. 2020	**Prospective, randomised, single-blind Treatment group** *N*=10, age=71, TSS=3.82 months **Control group** *N*=10, age=67.1, TSS= 5.33 months	Multisensory treatment: Bimodal audio-visual stimulation of visual field using visual field trainer Control group: Prism adaptation (goggles causing 10° rightward shift of the visual field) whilst doing a variety of 12 activities Both—10 working days, 2×20 min sessions	Star cancellation Bell cancellation Letter cancellation Line bisection Five element complex drawing Sentence reading Personal neglect Catherine Bergego Scale Timepoints: ×2 baseline T1 (day 7—1 week before starting) and T2 (day 1—on day of 1st treatment, prior to starting), T3 (day 6—end of 1st week of training and before starting training on the 6th day, T4 (day 11—end of second week of treatment). Subgroup had 1 month follow-up	**Star cancellation** Between group mean difference = 46% Cohen’s *d* = 0.71. (medium)
*Robot-assisted therapy*	Park et al. 2021	**Assessor blinded, randomised controlled Experimental group** *N*=12, age=69.08, TSS 9.5 months **Control group** *N*=12, age=71.58, TSS 9.08 months	Both groups did conventional treatments such as visual scanning training using prism and vibration on the left neck extensors and compensatory approaches. Experimental group did 20 sessions (5 days a week for 4 weeks) of robot-assisted hand training using the Amadeo Robotic device, each session lasting 30 min	Line bisection test Albert test Catherine Bergego Scale Timepoints: 1 pre and 1 post	**Catherine Bergego Scale** Within subject results Experiment group difference = −4.92 points on the CBS Control first group difference = −1.25 points on the CBS Between group Cohen’s *d* = 0.72
*Robot-assisted therapy*	Karner et al. 2019	**Assessor blinded, randomised controlled PARO group** *N*=21, age=74.21, TSS= 49 days **Control group** *N*=18, age=73.34, TSS= 55 days	Patients were treated for 2 weeks on 3 days per week, resulting in six sessions per patient. The duration of the individual intervention, including data collection, was 30 minutes, where the PARO robot was placed on the neglected side and patient had to grasp it. As control intervention, patients were read aloud from a book for the same time as the PARO intervention.	1°: CATS test Line bisection test Scores of independence Index for neurological and geriatric Rehabilitation Timepoints: T0 (baseline), T1 (after the 2 weeks of interventions), T2 (after an additional 2 weeks as a follow-up)	**CATS test** Between group effect size: T0 to T1 Cohen’s *d* = 0.70 (medium) T0 to T2 Cohen’s *d* = 0.99 (large)
*Robot-assisted therapy*	Chen et al. 2021	**Assessor-blinded, prospective, pilot randomised controlled trial Therapy group** *N*=10, age=46.2, TSS=97 days **Control group** *N*=10, age=48.6, TSS=86.4 days	Therapy group: 15-min passive session (with the exoskeleton making movements in a 3D trajectory) and a 30-min assist-as-need mode (patients played games with audiovisual feedback) Control group: Visual scanning therapy, passive upper limb range of movement exercises and perceptual retraining. Total dose was 45 min daily, 5 days a week for 4 weeks	Behavioural Inattention Test Catherine Bergego Scale	**Behavioural inattention test** Between group difference = +7.7 Cohen’s *d* = 0.24 (small) **Catherine Bergego Scale** No significant between group effect.
*Home-based visuomotor feedback training*	Rossit et al. 2017	**Single-blind, controlled prospective study Intervention group** *N* = 9, age = 65.6, TSS= 3.1 months **Control group** *N* = 9, age = 64.9, TSS= 3.2 months	Training delivered for two sessions of 30 min each by an experimenter and then patients self-administered it for 10 sessions over 2 weeks, asked to pick a rod at its midpoint versus at a corner by the control group	Behavioural Inattention Test (BIT) Line bisection Balloons test Landmark task Room description task Subjective straight-ahead pointing task Stroke Impact Scale Timepoints: At baseline, after 2 experimenter-led sessions, after 10 self-led sessions, follow-up at 4 months	**Behavioural Inattention Test score** *Intervention group* No significant between group effect.
*Non-invasive brain stimulation: rTMS theta burst*	Nyffeler et al. 2019	**Randomised, double-blind, sham-controlled 8cTBS group** *N* = 10, age=67.8, TSS=26.8 days **16cTBS group***N* = 10, age=74.3, TSS= 22.9 days **Sham group** *N* = 10, age=70.6, TSS= 25.8 days	3 groups: (1) Sham group (2) Continuous theta burst, 8cTBS trains (3) Continuous theta burst, 16cTBS trains	Catherine Bergego ScaleTimepoints: T0 (first week after admission to the clinic for stimulation), T1 (in the last week before discharge) and T2 (at follow-up 3 months later)	**Catherine Bergego Scale** Compared to sham stimulation, CBS score lower for both groups (no significant difference between 8c and 16c) Average mean difference −3.75 Average Cohen’s *d* = 0.74 (medium)
*Non-invasive brain stimulation: rTMS theta burst*	Vatanparasti et al. 2019	**Single-blinded, randomised controlled PA+cTBS group** *N*=7, age=67.5 **PA+sham group** *N*=7, age=65.5	Intervention group: Prism adaptation and cTBS over the left posterior parietal cortex for 10 sessions a day, for 2 weeks Control group: Prism adaptation with a sham TMS	Star cancellation test Line bisection task Figure copying test Clock drawing task Timepoints: At baseline and at the end of the 2 weeks	**Star cancellation test** No significant between group effect.
*Non-invasive brain stimulation: tDCS*	Gorsler et al. 2022	**Proof-of-principle, randomised double-blind sham-controlled, cross-over design** *N*=11, age=71, TSS=32 days	Four factorialised treatment sessions: active vs. sham crossed with unilateral vs. bilateral tDCS over the parietal region. 48-h wash-out phase between blocks. 4 sessions (each session separated by 2 days) of 20 min of tDCS or 30 seconds of sham stimulation given whilst patients did a 20-min computerized visual exploration or saccadic eye movement training task	Centre of cancellation index from the Bell’s testTimepoints: T1 (screening), T2 (baseline) and T3 (after completion of all 4 sessions)	**Centre of cancellation** No significant between group effect.
*Non-invasive brain stimulation: tDCS*	Learmonth et al. 2021	**Prospective randomised open blinded end-point feasibility trial Behavioural training** *N*=6, age=66.8, TSS=376 days **tDCS** *N*=6, age=66, TSS=469.3 days **Combined intervention** *N*=6, age=70.5, TSS=390.5 days **Control group** *N*=6, age=60.5, TSS=583.3 days	4 groups: (1) Behavioural training: Picking up and balancing wooden rods at the mid-point (2) tDCS (3) Combination of both (4) Control group: Picking up a wooden rod at its rightmost end 10 intervention sessions, with stimulation or training delivered for 15 min (overall target period 3 weeks)	Primary outcomes: Rate of recruitment Retention Compliance Secondary outcomes: Line bisection test Behavioural inattention test Timepoints: T1 (at baseline), T2 (after 3 weeks) and T3 (at 6 month follow-up)	**Behavioural inattention test (raw means)** *Behavioural training* T1=115, T2=130.4, T3=123 *tDCS* T1=105.17, T2=119.33, T3=136.20 *Combined intervention* T1=103.00, T2=126.20, T3=130.33 *Control group* T1=123.67, T2=130.20, T3=135.00
*Non-invasive brain stimulation: tACS*	Schuhmann et al. 2022	**Proof-of-concept, within-subject, placebo-controlled** *N*=13, age=57.8, TSS=87.4 days	All subjects underwent one session each of 10-Hz alpha stimulation, and the sham stimulation whilst doing a computerised visual detection task lasting 10 min, Bell’s test and line bisection task 2 separate sessions (one of 10-Hz alpha stimulation, and one of sham stimulation) with at least 1 day between sessions, stimulation lasting for a maximum of 30 min	Computerised visual detection task: involves assessing unilateral neglect and extinction by presenting Gabor patches just above individualised detection thresholds. Timepoints: 3 tasks done before, during and after each stimulation session	**Computerised visual detection task:** Average mean difference 4.1/40 trials Average Cohen’s *d* = 0.92 (large)
*Drug trials*	Luauté et al. 2018	**Double-blind, group randomised**: Stratified by recruitment centre and severity of neglect **Methylphendate group:** *N* = 13; age = 59; TSS = 3.7mo; severity = M9, S4. **Placebo group:** *N* = 8; age = 56; TSS = 5.2mo; severity = M4, S4.	Both groups received **prism training** (10° rightward shift) 5 sessions of 50 pointing responses to visual targets (2–5min). **Methylphendate group**: 10mg BD PO for 5 days.	1°: CBS 2°: star cancellation, FIM (functional independence measure). Time points: 2 pre and 3 post (immediate, 7days and 30days post)	**Catherine Bergego Scale** Group×time interaction (*p*=0.0204) Group difference = +3.7 on CBS Cohen’s *d* = 0.33 (small)
	Dalmaijer et al. 2018	**Double-blind, within-subject, order randomised, cross-over design:** *N* = 13; age = 63; TSS = 12.5mo; severity = M9, S4.	Subjects received a single dose of **Guanfacine** 2mg and a single placebo on day 2/day 4 of the study.	1°: Cancellation test 2°: sustained attention and working memory test. Time points: days 1 (pre), 3 and 5 (post)	**Cancellation test** (no. of targets): drug vs. placebo (*p*=0.013) Drug difference = +5 targets (out of 64) Cohen’s *d* = 0.34* (small)
	Swayne et al. 2022	**Case series, single-case, open label experimental design** (ABA): *N* = 10; age = 56; TSS = 8.6mo; severity = M7, S3.	*N* = 3 **rotigotine** transdermal 4 mg/24 h. *N* = 7 **co-careldopa**, 100/25 mg TDS. Off-on-off design with each block lasting a week.	1°: Cancellation test % of stars cancelled to affected side.	**Cancellation test** (% of targets): drug vs. placebo (*p*=0.004) Drug difference = +27% targets on affected side Cohen’s *d* = 2.1* (large)

*BD* twice a day, *CBS* Catherine Bergego Scale, *M* moderate neglect, *mo* months, *N* number, *PO* by mouth, *S* severe neglect, *TDS* three times a day, *TSS* time since stroke, *NB* acute < 1month post-stroke; post acute = >1 month but <3 months, (likely on a rehab unit); chronic = >3months (likely in the community). *Several studies have calculated Cohen’s *d* using a within-subject method. In order to make these more comparable with between-group studies, we have recalculated, treating data as if from different groups

### Sensory Stimulation

#### Eye Movement–Based Therapies

Eye movements are closely linked to spatial attention, both at a behavioural and an anatomical level [4]. The superior colliculus acts as a conduit of sensory and motor signals to the cortical and subcortical areas responsible for eye movement control [5]. Many cortical regions in the dorsal frontal and parietal areas are involved, with a rightward hemisphere bias, in the control of attention [6]. Deficits of function in the superior colliculus can be compensated for by the frontal eye fields, and vice versa [7, 8]. This close relationship between spatial attention and eye movements forms the basis for visually based approaches.

In neuro-rehabilitation, both saccadic and smooth pursuit eye movement–based training has been trialled. In visual scanning training, patients have to find static targets presented across the visual field using voluntary saccades. In contrast, smooth pursuit eye movement training involves following moving targets that ‘drag’ the patient’s attention towards the neglected side.

Elshout et al. [9] undertook a proof-of-concept, single-blinded, group randomised controlled trial, comparing congruent movement training to visual scanning training alone in patients in the chronic phase. Stimuli (filled, coloured circles) were presented on a 2D screen. The congruent movement training group had to find certain circles and touch them whilst the control group only made eye movements and reported how many circles of a certain type that they could see. They practiced ten 30-min sessions for a total of 5 h. The researchers, rather unusually, created a composite outcome score from two cancellation tests and the CBS. There was a statistically significant difference between the groups on this measure although it was in part driven by the visual scanning group’s score getting worse. The effect size was medium, giving some support to the idea that reaching with both a limb and eyes is superior to reaching with eyes only.

Yasuda et al. [10] trialled a single-shot (30min) immersive virtual reality (iVR) intervention using a within-subject, order randomised, pre-post design with no control task or blinding. Ten patients in the chronic phase took part, performing both near (a reaching task) and far space (a visual search task) training. They used the Behavioural Inattention Test (BIT) as their main outcome measure. Rather oddly, they performed no statistical tests of the interaction between space (near vs. far) and time (pre vs. post), instead reporting that the BIT increased significantly for the far training only and not near training. Even taken at face value, these results provide only weak evidence that visual scanning training may be beneficial. The VR was well tolerated by patients.

Choi et al. [11] conducted a single-blinded randomised controlled trial of 24 patients in the chronic phase. The therapy group performed 10 different tasks on the Oculus Rift iVR device at a rate of three 30-min sessions a week for 4 weeks. The control group underwent conventional unilateral spatial neglect training for the same time period. After training, the mean CBS scores between the two groups did not significantly differ. The authors chose to focus on a bespoke outcome measure that did differ between the groups, the Motor Visual Perception Test–Vertical version. It comprises five impairment-based tests, but removes any horizontal bias, so the iVR did not influence lateralised attention at all.

Eye movement–based therapies remain one of the most popular approaches to treating visuospatial inattention. It is a bit surprising that these three recent studies all relied on inducing voluntary guided saccades as smooth pursuit methods have been shown to be more effective [12]. iVR seems a very promising technique that can treat patients with stimuli not limited to the width of a computer screen, although the two studies reviewed here were not particularly effective. Other work suggests that it is well tolerated, even in the acute phase [13].

#### Auditory Spatial Cueing

Inattention can be expressed in any of the main sensory domains [14, 15], with the corollary being that these domains can be used as channels to stimulate lateralised attention [16, 17].

Auditory stimulation, particularly in the form of pleasant music, has been shown to activate the striatum, anterior cingulate cortex and the orbitofrontal cortex, areas that play a role in visual attention, emotion and cognition [18–20]. Coupling auditory and visual stimuli so that they appear to emanate from the same position in neglected space has been shown to create an improvement in visual detection in patients with hemispatial inattention [21, 22].

Kaufmann et al. [23] conducted a proof-of-concept, controlled trial design using a novel dynamic auditory technique, with stereo sound moving from the right to the left (neglected) side. They undertook two separate experiments on two independent groups of patients in the acute phase, looking at the immediate effects of spatial auditory stimulation lasting for 10 min in experiment 1, and the after-effects (1 and 3 h) in experiment 2. The first experiment was a cross-over design with a block of auditory spatial cueing (where music appeared to travel horizontally from the right to the left) which was compared with a control block where musical stimulation was identical bilaterally (no illusory horizontal movement). A cancellation test was used as the outcome measure. They found a significant improvement with auditory spatial cueing, and a large effect size of 0.85. Experiment 2 was group randomised. Participants were randomly assigned to either the spatial auditory cueing or control condition. Free visual exploration (a sensitive impairment-based measure) was recorded at baseline and at 1 and 3 h post exposure. Whilst they found no significant differences in mean gaze position between both groups at the 1-h timepoint, they did find a significant difference at the 3-h timepoint with spatial auditory cueing leading to reduced hemispatial inattention (eta square = 0.039) indicating a small after-effect. They posited that spatial auditory cueing has a similar bottom-up effect as smooth pursuit eye movement training, and their results certainly encourage using spatially dynamic auditory stimulation in future mutli-sensory studies, as opposed to simple music/white noise alone.

Schenke et al. [24] carried out two pilot studies in the post-acute phase. The first assessed the effects of auditory stimulation with dynamic cueing, whilst the second investigated whether the addition of auditory cueing to optokinetic stimulation was beneficial. Study 1 used a group randomised design, with patients receiving 3 weeks of daily 30-minute sessions listening to music that appeared to travel towards the affected side. The control group received neuropsychological sessions. Line bisection was the primary outcome measure. Both groups improved, but there was a significant difference favouring the auditory stimulation group with a small effect size (0.38). In the second study, eight new patients received fifteen 30-minute sessions over 3 weeks, where optokinetic stimulation and a spatial auditory cueing were combined. A visual scanning test was used as the outcome measure. The within-group effect size was huge (2.25), further supporting the use of dynamic auditory cueing as a complimentary combination tool for existing therapies, although the lack of a control group in the post-acute phase means that a reasonable portion of this effect was likely due to time effects alone.

Zigiotto et al. [25] undertook a prospective, randomised, single-blinded study comparing audio-visual stimulus with prism adaptation. The audio-visual treatment group received twice daily, 20-min sessions over 10 days in the form of a training board with light-emitting diodes, and loudspeakers emitting sound. Patients were asked to follow a visual target that appeared simultaneously with a sound in the same location. The prism adaptation control group did an equal number of sessions, performing a range of 12 activities using goggles that caused a 10° rightward shift of their visual field. On star cancellation, both groups improved with time but there was a significant time×group interaction with a between group difference in favour of the multisensory group with a medium effect size. Both groups saw a reduction in CBS scores over time, with no significant time×group interaction reported.

Dynamic auditory stimulation is a very promising addition to the therapeutic arsenal. Like other sensory stimulations that re-orient attention (e.g. caloric), it seems to have a reasonable effect in the short term. It will be interesting to see if these effects can be made to persist, perhaps by pairing the stimulation with more conventional, therapist-delivered sessions. The approach is low-tech and portable so will hopefully be included in future trials.

#### Robot-Assisted Therapy and Sensory Feedback

Passive and active contralesional upper limb movements, even in the absence of intentional motor programming, such as with functional electrical stimulation, have been noted to create improvements in hemispatial inattention [26, 27]. The mechanism presumably involves attentional orientation in response to sensory (light touch and joint position sense) feedback from the affected limb.

Park et al. [28] conducted an assessor-blinded, randomised controlled trial to look at the effects of robot-assisted left-hand training in older adults in the chronic phase. The experimental group performed twenty 30-minute sessions, 5 days a week for 4 weeks, of training with the Amadeo Robotic device, which provides motion of one or all five fingers through passive rotational joints that cover the fingers’ workspace. The control group performed conventional treatments such as visual scanning training using prism and vibration on the left neck extensors and compensatory approaches. Outcome measures included the line bisection test and the CBS. On the CBS, the experimental group showed a mean raw score difference of −4.9 points, above the MICD. Comparison with the control group revealed a medium effect size of 0.72 favouring the use of robotic therapy.

Karner et al. [29] used an assessor-blinded, randomised controlled trial design to evaluate the effects of a robotic baby seal called PARO, capable of moving, producing sounds and reacting to speech and touch. Patients in the sub-acute phase received a total of six 30-minute sessions over 2 weeks, during which they had to pay attention to PARO, who would then move further into the affected hemi-space. The control group were given a book to hold. They were read aloud to for 30 minutes. The primary outcome measure was a cancellation task. The PARO group did significantly better on this test than the control group both at the immediate post therapy time point (medium effect size) and 2 weeks later (large effect size).

Chen et al. [30] undertook an assessor-blinded randomised controlled trial to test the effects of exoskeleton-driven robot-assisted arm training. Patients were at the sub-acute/chronic phase border. Those in the therapy arm had a 15-minute passive session (with the exoskeleton making movements in a 3D trajectory) and a 30-minute assist-as-need mode (patients played games with audiovisual feedback). Those in the control group did visual scanning therapy, passive upper limb range of movement exercises and perceptual retraining. The total dose was 45 minutes daily, 5 days a week for 4 weeks. Outcome measures included the BIT and the CBS, with the former showing a small but significant difference that favoured the robot, and the latter showing none.

Rossit et al. [31] tested the efficacy of home-based visuomotor feedback training in a single-blinded, controlled, prospective study of patients just in the sub-acute/chronic phase. The intervention group had two experimenter-led sessions followed by 10 self-administered sessions at home over 2 weeks, learning a task that required them to pick up a rod at its midpoint versus the control group who were asked to pick it up at the end. They used the BIT as their outcome measure. Both the control and intervention groups showed large improvements in their mean BIT score, and although the experimental group improved more numerically, the effect was not statistically significant.

The evidence from robotic studies is promising. Those that induce passive movements (Park and Chen) seem to work well as do those requiring interaction (PARO). Whilst a more expensive approach, the possibility of addressing both upper limb hemiparesis and lateralised inattention at the same time is enticing.

#### Mirror and Prism Therapies

There have been many studies using these two techniques which rely on altering visual inputs in order to redirect attention to the neglected side. Space issues preclude formal assessment of individual papers, but two recent meta-analyses summarise the current evidence well, particularly Székely et al. on the use of prisms [32]. Zhang et al. performed a formal meta-analysis of five studies of mirror therapy published over the last 8 years. When undergoing mirror therapy, patients practice attending to their neglected side by looking at a mirror placed perpendicularly to them and just off-centre. This reflects voluntary movements that they make with their unaffected upper limb, giving the illusion that the movements are taking place on the neglected side. The premise is that whilst sensory feedback from their unaffected limb might drive attention away from the affected side, the fact that they are staring into affected space and experience the illusion of seeing their affected arm move is a more powerful lateralising attentional stimulus. Studies are usually carried out with patients in the sub-acute phase receiving in-patient rehabilitation. Group randomisation is used with either care-as-usual or sham therapy consisting of using a non-reflective surface for the control group. Therapy sessions are typically led by a physiotherapist, last 20–60 minutes and are given at the rate of ~five sessions a week for 3–6 weeks. Zhang et al. found large effects on impairment-based outcomes (standardised mean difference of 1.62) and functional outcomes (2.09), suggesting that the approach is effective; however, they caution that the studies all suffer from potential performance bias (participants unblinded) and there were not enough studies included to rule out publication bias.

The Székely et al. meta-analysis is the most comprehensive and definitive to date, covering 16 trials from over 20 years of work. Prisms were used by Hermann von Helmholtz in the late nineteenth century as a demonstration of (transient) perceptual leaning; it was not until the late 1990s that they were used to treat visuospatial inattention. Prism adaptation has three phases. In the pre-exposure phase, the patient points to a visual target (usually accurately). In the exposure phase, patients are fitted with prism lenses that laterally displace the visual field *away* from the neglected side (typically by 10°). They now have to point at the same targets but will miss them in the direction of the displaced image. The therapeutic component occurs in this phase as they must learn to point more *towards* the neglected side in order to reach the target accurately. In the post-exposure phase, the prisms are removed and the patient will now point with an error biased *towards* the neglected side. These after-effects soon wear off, but the theory is that the procedure induces a more lasting effect of ‘spatial realignment’. The parieto-cerebellar network likely mediates this effect [33].

Across the 16 studies analysed, there was wide variability in the time since stroke from the first 2 weeks up to several years. Length of treatment was more standardised across studies at ~14 days but the sessions were short, with the number of pointing movements during each adaptation session being no more than 100 and the total number of sessions (across all training days) averaging at only 10. The studies were judged to have a high risk of bias using the revised Cochrane criteria, although these criteria are not designed with complex interventions in mind. They found no significant publication bias. On the impairment side, the standardised mean difference was 0.24 but the 95% CI included the line of null effect. On the CBS outcome, the result was similar, a standardised mean difference of 0.26 that could not exclude a null effect.

Contrasting these two approaches, it seems that mirrors are more promising than prisms, although there is likely more bias in the meta-analysis of the mirror studies. If it were the case though, what might be the explanation? In terms of what happens during therapy, we have three observations: firstly, mirror therapy studies employ a considerably higher dose measured as time-on-task than prism therapies do; secondly, in mirror therapy, the patient spends all their time attending visually to the affected side, whilst in prism therapy the exposure phase involves shifting visual attention away from the affected side and all three phases generally involve patients pointing to both the affected and unaffected sides. Lastly, mirror therapy studies have mostly been undertaken in patients in the sub-acute rehabilitative phase, when they are interacting with therapists as well as having their reorienting therapy. Many of the prism therapy studies are done in the chronic phase where the patients may well be having little or no ongoing therapist-delivered rehabilitation.

### Non-invasive Brain Stimulation

Kinsbourne proposed the Rivalry Theory in 1981, whereby both visual hemifields receive attentional input from the right hemisphere, whilst the left hemisphere only directs attention towards the right visual field, explaining why right hemispheric lesions cause inattention more commonly and more profoundly. He also suggested that the hemispheres compete with each other, with excitatory and inhibitory intercallosal reciprocation between hemispheres to allow one side to be activated when directing attention towards the contralateral visual hemifield [34–36]. This opens up the possibility of using non-invasive brain stimulation as a treatment modality in inattention, ‘rebalancing’ disrupted patterns of resting activity (too much on the left, not enough on the right). In recent years, repetitive transcranial magnetic stimulation (rTMS using a theta burst stimulation (TBS)), transcranial direct current stimulation (tDCS) and transcranial alternating current stimulation (tACS) have all been trialled.

#### rTMS—Theta Burst

Nyffeler et al. [37] studied 60 patients in the sub-acute phase with a randomised, double-blind, sham-controlled design. The 30 patients in the rTMS group were randomised into three groups: 8cTBS, 16cTBS or sham. The other 30 patients were controls (no TMS), but oddly their data never featured in the main analyses, so it is not clear why they were also not randomised into one of the three TMS groups. The 8cTBS group received eight sessions of theta burst stimulation (an inhibitory repetitive transcranial magnetic stimulation protocol) over the left posterior parietal cortex over 2 days, whilst the 16cTBS group got double the dose over the same time period. CBS was the primary outcome measure. The authors reported a significant improvement in the CBS after both 8cTBS and 16cTDS compared to sham stimulation with a medium effect size of 0.74 and a change in the CBS of −3.75 which is just under the MICD. No further improvement or decrement was noted at 3 months follow-up. These results help establish that a TBS over 2 days may well be beneficial, although the change in CBS was borderline in terms of clinical relevance. There was no obvious benefit of the higher dose 16cTBS protocol.

Vatanparasti et al. [38] used a single-blinded, randomised controlled trial design to assess the effects of combining continuous TBS with prism adaptation. Only 14 patients in the subacute/chronic phase were randomised into either the intervention group, who received prism adaptation and cTBS over the left posterior parietal cortex 10 sessions a day for 2 weeks, or the control group, who had prism adaptation and sham TMS. Star cancellation was the primary outcome measure but there was no significant between-group effect.

#### tDCS

Gorsler et al. [39] executed a well-designed proof-of-principle, randomised double-blind sham-controlled study with a cross-over design to assess the differences between unilateral and bilateral tDCS protocols. Patients at the acute/sub-acute boundary received four randomised treatment sessions, during which one of the two active or sham protocols were applied whilst having neglect therapy, with a 48-h wash-out phase between cross-over. The Bells cancellation test was the primary outcome but there were no significant between-group effects.

Learmonth et al. [40] conducted a group-randomised open, blinded end-point feasibility trial to compare behavioural training (picking up and balancing wooden rods at the mid-point), tDCS, and a combination of both compared to a control group (picking up a wooden rod at its rightmost end). Twenty-four participants in the chronic phase (so only six in each group) received 10 sessions of an hour each over 3 weeks across four hospitals in the Glasgow area. The BIT was the main outcome, but due to a low recruitment rate, statistical analyses were not carried out. They concluded that a larger scale trial would not be feasible as too many patients were excluded due to significant co-morbidity, preventing participants from undergoing the required 10 intervention sessions.

#### tACS

Schuhmann et al. [41] undertook a within-subject, placebo-controlled study, to look at the effects of transcranial alternating current stimulation on 16 patients in the chronic phase. They applied sham and high definition tACS (HD-tACS) over the contralesional posterior parietal cortex in two separate sessions on two different days with at least 1 day between them. They used a bespoke, computerised visual detection task which assessed unilateral neglect and extinction by presenting Gabor patches just above individualised detection thresholds. They found that after HD-tACS patients were better at detecting targets (~+10%) in their affected hemifield.

Whilst rooted in the Kinsbourne Rivalry Theory, trials of brain stimulation have generally been less successful than other approaches. TMS has a stronger evidence-base than the tDCS, perhaps because the former is considered a neuro stimulator and the latter a neuro modulator, with the implication that tDCS needs to be paired with some form of sensory stimulation or task to be effective. Whilst all studies have to deal with the hard-to-model effects of differential damage across the spatial attentional system caused by stroke, given the focal nature of these therapies, these effects are likely amplified. Thus, lesion-based individual differences should inform future study designs.

### Drug Therapy

Drug studies in humans were first attempted in the 1980s following on from animal lesion-based studies that suggested dopaminergic depletion could cause visuospatial neglect. Dopamine agonists were the first to be used (bromocriptine) and dopaminergic drugs remain the main class to be trialled in recent years, either as a pro-drug (l-Dopa), an agonist (rotigotine) or a reuptake inhibitor (methylphenidate). Guanfacine, a noradrenergic alpha-2A agonist, has also been utilised.

Luauté et al. [42] carried out a well-designed study investigating methylphenidate’s effects on hemispatial inattention. The drug and placebo groups both received prism training across five sessions. There was a significant time by group interaction favouring the methylphenidate group on their primary outcome measure, the CBS. The authors did not carry out any post hoc tests to see which time points were driving the effect, but eyeballing the data suggests that a small gain was made immediately post therapy ~1.2 points on average with further gains at 30 days when compared with the placebo group. The unstandardised change in score between the groups was small (−3.7 points). This is also reflected in the small Cohen’s *d* (0.33). The authors speculated that the drug effect was independent from that of the prism training.

Dalmaijer et al. [43] used a simple, one-dose, cross-over trial design to look at the effects of guanfacine in 13 patients in the chronic phase. Their impairment-based outcome was a touchscreen cancellation task. Because drug effects have been shown to affect both sustained attention and spatial working memory, the authors measured these at multiple time points too. They used an interesting additional statistical approach, calculating Bayes Factors, which enabled them to estimate the probability of the null hypothesis being true. They found that guanfacine significantly improved target cancellation scores (small effect size), but that there was no lateralised effect. Their Bayesian approach allowed them to infer from their null effects that the action of guanfacine was not via enhanced spatial working memory, response times or executive control of searching, but could not adjudicate one way or the other on whether it was affecting sustained attention.

Swayne et al. [44] studied the effects of 1 week of either rotigotine or l-dopa in an open-label, within-subject, A-B-A design. Patients were on-drug during the middle week which was compared with the 2 off-drug weeks either side. They found a large effect at the group level which must be tempered by the non-blinded (open label) nature of the study. There was, unsurprisingly, variation within the group, and when a binarised ‘overall clinical perspective’ judgement was made, only 6/10 were considered to be responders. The lack of detailed neuropsychometric outcomes meant that it was not possible to adjudicate as to the possible cognitive mechanism(s) underlying the improved target detection. The authors suggest that the best way to tackle heterogeneity issues (responders and non-responders) is via well conducted (and blinded) N-of-1 studies, rather than taking a group-randomised approach.

In common with many of the therapeutic approaches to hemispatial inattention, drug studies suffer from low numbers of patients being treated and the potential for bias affecting published results. Despite this, drug approaches seem promising. Theoretically, they are the easiest intervention to control for in terms of having a placebo. The cognitive mechanisms of drug therapy are still unclear, with rival theories positing either a direct effect on lateralised attention or an effect on non-spatial attention or even arousal. The Dalmaijer et al. study paves the way for addressing this by having tests of key cognitive components (sustained attention, working memory and executive control of visual search) alongside the more standard impairment and function-based outcomes. Employing Bayesian statistics to help adjudicate null findings is also a good practice, and, with greater numbers of patients, will likely help resolve these issues.

Dose and timing factors remain unclear, but in the post-acute phase, and if the patients are still in hospital with access to therapist-delivered neurological rehabilitation, it makes clinical sense to have therapy blocks of at least a week. However, the greatest barrier to clinical translation is between-subject heterogeneity. What factors, anatomical or behavioural, that feed into this remain unclear. We agree with Swayne et al. that designing studies so that statistical evaluation can be carried out on individuals when both on and off drug (preferably with more than one cycle of this, so ABAB) as single-case experimental designs (SCEDs) is probably the best way forward. These trial designs often still allow for a between-subject or group effect analysis via either a standard ANOVA or a multi-SCED approach.

## Conclusions

The field of rehabilitation of hemispatial inattention continues to be a lively one, filled with many innovative approaches. In general, studies are, and will continue to be, affected by the small number of participants. This is not an issue in terms of estimating effect sizes, as there is little point conducting large-scale trials capable of detecting small effects as these will not make their way into clinical practice. Rather, the issue pertains to managing heterogeneity. Patients vary greatly in the severity of their symptoms, how quickly they recover with the passage of time and the distribution of their lesions. As such, we advocate the following three approaches.

Firstly, future studies should consider implementing SCED designs. These can be constructed such that a group comparison can still be made if the intervention is allocated at a group level. If therapy and control blocks can be randomly allocated to each individual, this is statistically more powerful. Because patients are rarely on a stable baseline, change scores across blocks can be used rather than absolute values as there is almost always a significant simple effect of time. SCED designs require multiple outcome measures be obtained. On the impairment side, this can be done with old-fashioned cancellation tests, but newer, more sensitive tests are available that have better reliability, are quick to use and can be delivered via a computer, e.g. free visual exploration [45].

Secondly, if the therapy is going to be group randomised, then minimisation or stratification procedures should be used to ensure that the groups do not become unbalanced on one or two key variables, such as severity or time since stroke.

Lastly, visuospatial inattention is a multi-sensory disorder. The behavioural interventions we use are complex by nature (which makes them hard to create sham therapies for) but we should consider pairing them up more, e.g. visual and auditory, limb movements and haptic. For drugs and brain stimulation approaches, these are likely to be more effective when paired with some form of sensory-based spatial retraining or ongoing therapist-delivered neurological rehabilitation.
